# Nerve Sparing Clitoroplasty via a Ventral Approach in a Child With Congenital Adrenal Hyperplasia: A Case Report and Review of the Surgical Technique

**DOI:** 10.7759/cureus.110826

**Published:** 2026-06-14

**Authors:** Biswajit Mishra, Suvendu Amitav, Deepak Kumar Sahu, Madan M Majhi

**Affiliations:** 1 Plastic Surgery, SCB Medical College and Hospital, Cuttack, IND; 2 Community Medicine, SCB Medical College and Hospital, Cuttack, IND

**Keywords:** clitoromegaly, clitoroplasty, congenital adrenal hyperplasia, feminizing genioplasty, nerve-sparing, ventral approach

## Abstract

Clitoromegaly secondary to congenital adrenal hyperplasia (CAH) poses a significant surgical challenge, requiring preservation of neurovascular integrity alongside satisfactory aesthetic restoration. The optimal timing and operative approach for feminizing genitoplasty remain subjects of ongoing debate. We report a six-year-old girl with classic CAH (Prader stage 3) who underwent nerve-sparing clitoroplasty via a ventral approach. The technique prioritised preservation of the urethral plate, dorsal neurovascular bundle, and clitoral glans. At the six-month follow-up, both the patient and family expressed high satisfaction with cosmetic and functional outcomes. Ventral nerve-sparing clitoroplasty offers reliable aesthetic results with preservation of neurovascular structures. Magnification loupes, meticulous dissection, and intraoperative papaverine irrigation minimise the risk of vasospasm and neurovascular injury. Multidisciplinary care and community-level awareness are essential for timely intervention.

## Introduction

Clitoromegaly in female children with congenital adrenal hyperplasia (CAH) due to 21-hydroxylase deficiency is among the most common indications for feminizing genitoplasty. The ideal timing of surgical correction remains a matter of debate. The 4th World Congress of the International Society of Hypospadias and Disorders of Sex Development Surgery, as well as the American Academy of Pediatrics, recommends intervention before two years of age to optimise anatomical and psychosocial outcomes [1-3].

Advocates of early surgery cite improved anatomical restoration, greater patient self-esteem, and reduced parental distress. Long-term data suggest that adult women who underwent childhood surgery tend to retrospectively endorse earlier intervention [4]. Conversely, critics emphasise the risk of diminished clitoral sensitivity, impaired orgasmic function, and the need for re-operation. Crucially, the child cannot participate in the decision-making process at an early age [5].

Current consensus favours a multidisciplinary approach with transparent family counselling, disclosing all risks and benefits, including the option of conservative management [6,7]. Although patients who underwent early correction have demonstrated improved self-confidence and body image, no definitive evidence has established the superiority of early over deferred surgery [8-10].

Herein, we report a case of childhood clitoroplasty performed using a ventral nerve-sparing approach, which is likely among the first such reports from this region of India. Patient satisfaction and surgical outcomes were assessed over a six-month follow-up period.

## Case presentation

A six-year-old child, raised as female, was referred to the Department of Plastic Surgery for evaluation of clitoral enlargement. She had initially presented to the Endocrinology Department with hyponatremia, hyperkalemia, and failure to thrive. Physical examination revealed a phallus-like structure, a single urogenital opening, and posterior labial fusion consistent with Prader stage 3 virilization, with no palpable gonads. Biochemical investigations demonstrated a basal 17-hydroxyprogesterone (17-OHP) exceeding 52 ng/mL and an 8:00 AM serum cortisol of 3.66 μg/dL.

**Figure 1:**
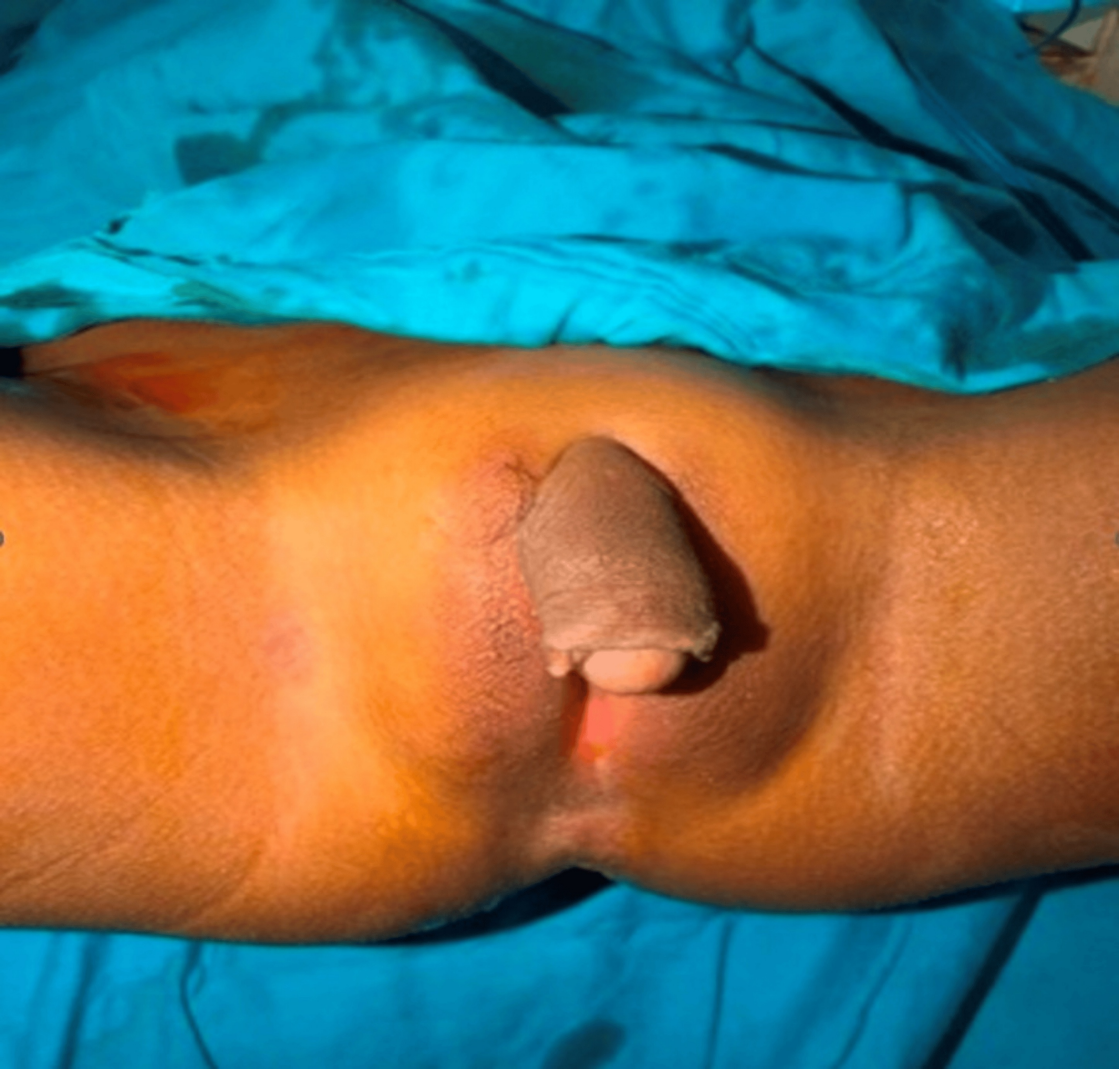
Preoperative appearance of clitoromegaly with phallus-like structure.

**Figure 2:**
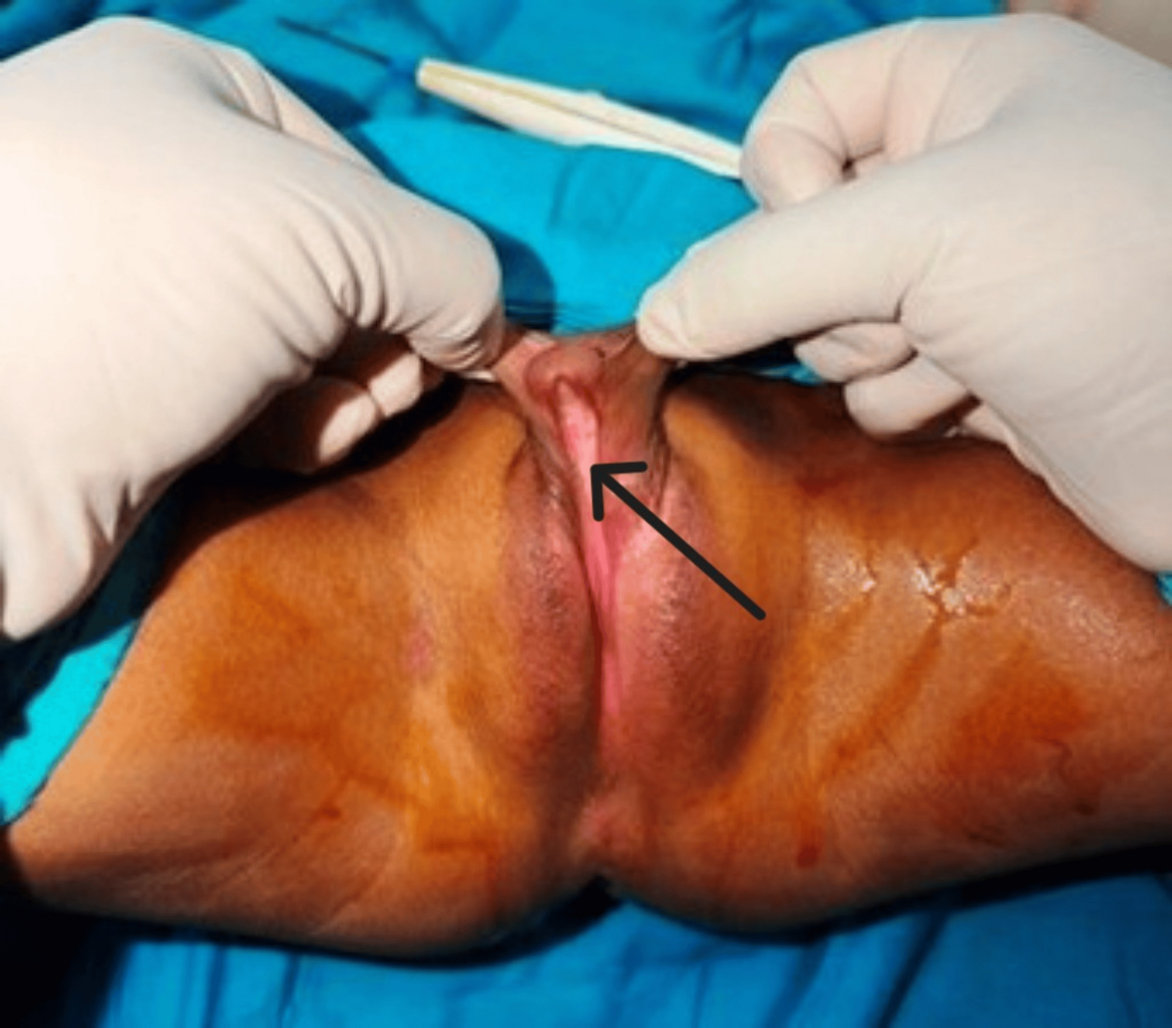
Ventral urethral opening extending from the tip of the clitoris to the perineum, resembling hypospadias. The Black arrow indicates the urethral opening extending from tip of clitoris to Perineum

Ultrasonography of the abdomen and pelvis confirmed normal Müllerian derivatives and absence of Wolffian structures. Integrating the clinical, biochemical, and radiological findings, a diagnosis of the classic salt-wasting variant of CAH was established. The patient had been on medical therapy for six months of age. At surgical assessment, the phallus-like structure displayed a ventral urethral opening extending from the tip of the glans to the perineum, resembling penoscrotal hypospadias in configuration (Figures 1-2).

Surgical technique

**Figure 3:**
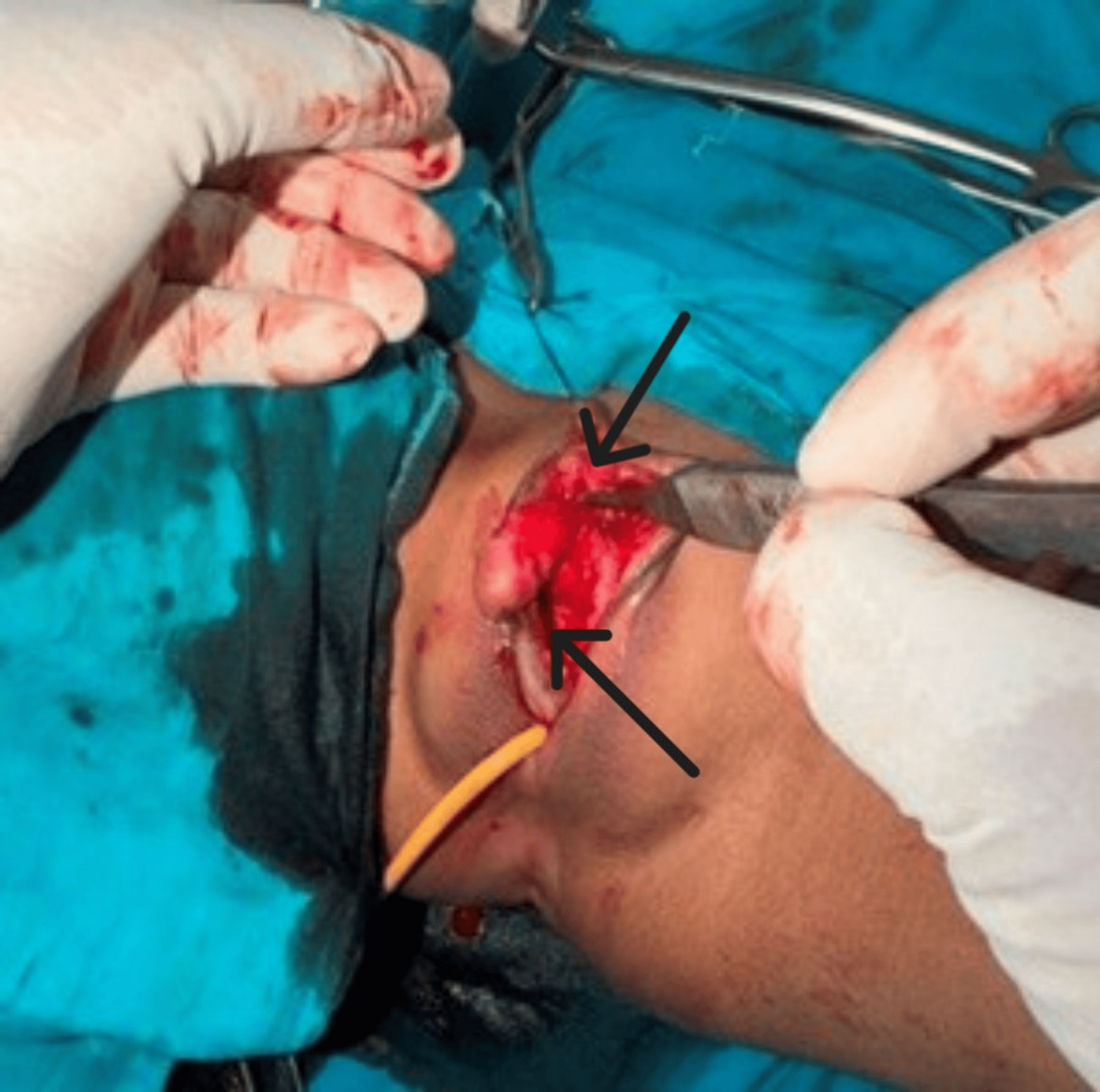
Intraoperative view following circumcoronal degloving to the level of the pubic symphysis. The urethral plate is intact and preserved. The upper black arrow indicates degloved skin dissection up to the pubic symphysis. The lower black arrow indicates the preserved urethral plate.

A circumcoronal incision was made, and the skin and dartos layer were degloved to the base of the phallus. Ventral incisions were then placed on either side of the existing urethral plate, followed by incision of Buck's fascia and the tunica albuginea. The corporal bodies were carefully dissected from within the tunica, with all dorsal tissue preserved to maintain the integrity of the neurovascular bundle (NVB). The corporal bodies and urethral plate were dissected proximally, separating them from the NVB to the level of the pubic symphysis; each corpus was transected proximally, preserving a 1.5-2 cm stump, which was oversewn with 3-0 Vicryl (Figure 3).

Glans reduction was performed by excising the ventral component. The residual ventral glans was sutured to the tunica at the level of the corporeal bifurcation, and the dorsal glans was anchored to the subcutaneous tissue at the pubic symphysis. Papaverine was applied topically to the neurovascular pedicle throughout the dissection to prevent vasospasm, and meticulous haemostasis was achieved under magnification loupes.

The patient was discharged on day five with an indwelling urethral catheter. Follow-up was conducted at two weeks, one month, three months, and six months postoperatively.

**Figure 4:**
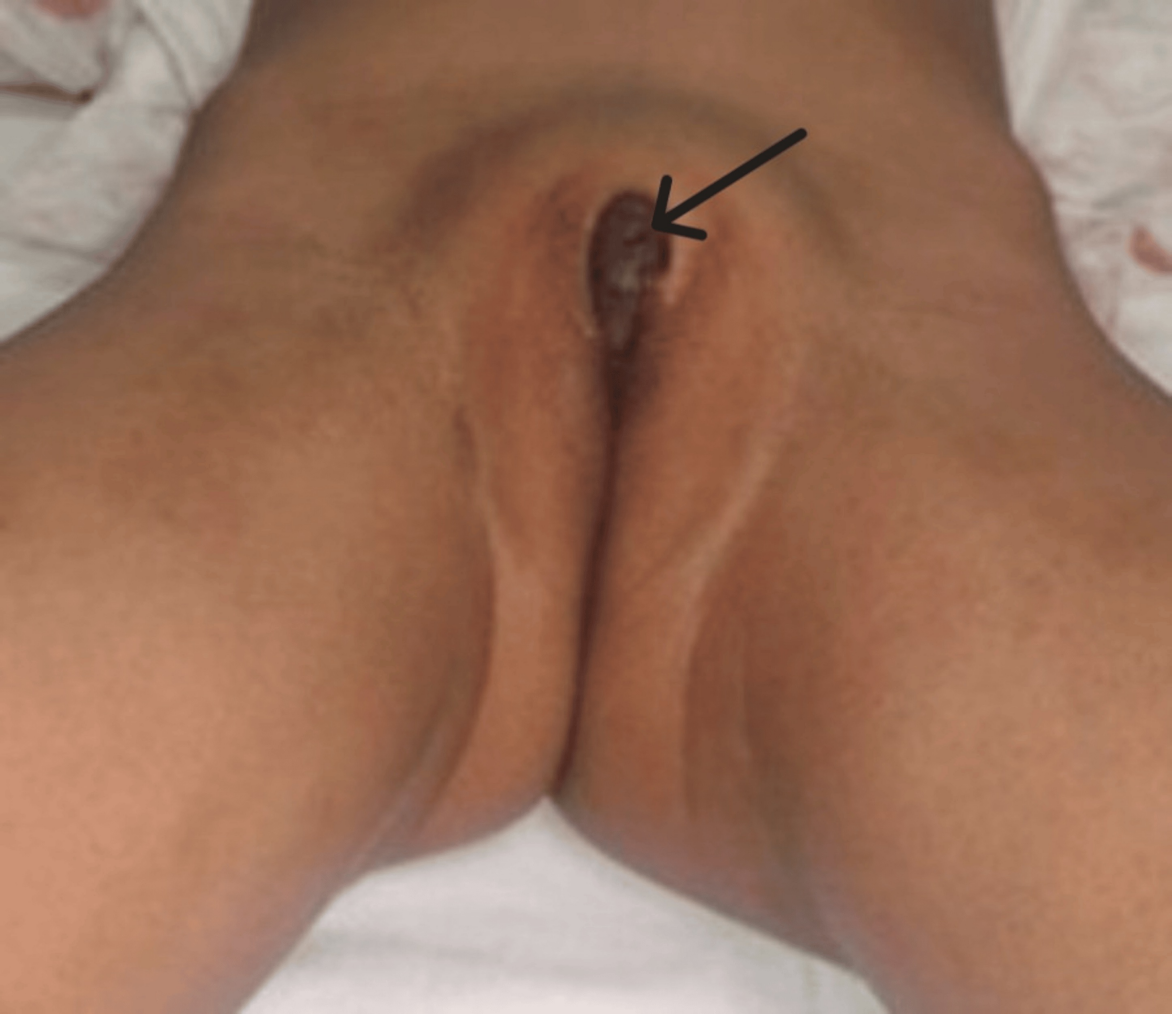
Six-month postoperative result. Oedema has fully resolved. The clitoral hood is well maintained with a satisfactory aesthetic appearance. The black arrow indicates well maintained clitoral hood.

Mild postoperative oedema was noted during the early recovery period and resolved completely by three weeks. There was no pain, wound dehiscence, or vascular complication. Clitoral sensation was clinically preserved on tactile stimulation at the three-month review. Both the patient and her parents expressed high satisfaction with the cosmetic and functional outcome at six-month follow-up (Figure 4).

## Discussion

The goals of feminizing genitoplasty in CAH are well established: to reconstruct the external genitalia with an appearance consistent with the assigned gender, to enable unobstructed urinary drainage, to permit unimpeded menstrual flow, and to preserve the potential for a satisfactory reproductive and sexual life in adulthood [1]. The surgical armamentarium encompasses clitoroplasty, vaginoplasty, and labioplasty, performed either as staged or single-session procedures.

The evolution of clitoral surgery spans nearly a century. Young first described the surgical management of clitoromegaly in 1934 [11]. Early approaches consisted of total clitorectomy, which resulted in irreversible loss of clitoral sensitivity, painful erections, and profoundly unsatisfactory functional outcomes, compelling factors that led to the eventual abandonment of ablative techniques.

In 1961, Lattimer introduced a more conservative strategy, advocating relocation of the clitoris beneath the pubic symphysis while preserving the glans and debulking the erectile tissue [12]. Randolph et al. subsequently refined this approach by resecting the exposed corporeal shaft while sparing the glans [13]. The landmark contribution of Kogan in 1983 was the subcutaneous reduction technique, which established preservation of the neurovascular bundle as the cornerstone of modern clitoroplasty [14].

The work of Newman et al., Crouch et al., and Yang et al. progressively reinforced the primacy of clitoral function in female sexuality, with each successive publication advocating more conservative dissection [8,15,16]. In 2007, Pippi Salle et al. introduced corporeal-sparing dismembered clitoroplasty (CSDC), in which the NVB and glans are freed from the corpora cavernosa [17]; the corpora are then divided longitudinally into two hemicorpora and transposed into dartos pouches within the labia majora. A notable advantage of this technique is the retention of erectile tissue for potential future phalloplasty in the estimated 5% of CAH patients who may subsequently identify as male [17].

An alternative paradigm, described by Hurwitz, involves excision of the corporeal erectile tissue with meticulous preservation of the dorsal neurovascular pedicle [18]. This approach has gained wide acceptance for its theoretical ability to maintain clitoral sensitivity through protection of the somatosensory and vascular supply to the glans. Currently, most surgeons excise erectile tissue while preserving the NVB, thereby creating a functional neoclitoris of reduced dimensions [9,19-22].

A critical intraoperative principle involves preservation of the tunica albuginea and Buck's fascia in the dorsal compartment following their release, alongside protection of the clitoral glans. Together, these steps minimise inadvertent injury to the dorsal NVB, damage to which may result in postoperative paraesthesia, ischaemia, or glans necrosis [14,23].

In the present case, a ventral approach was employed as described by Patil et al. [21] and refined by Yang et al. [16] and Sönmezer [23]. Unlike the CSDC technique, the corpora were not buried, as burial within the labia has been associated with painful erections [17,20,24]. The ventral incision afforded direct access to the urethral plate, enabling its dissection and preservation in the context of anomalous urethral anatomy resembling proximal hypospadias as an anatomical configuration not previously described in comparable published reports [20,21].

Kujur et al. [20] employed a dorsal approach to preserve the NVB in idiopathic clitoromegaly; in that series, as in the report by Patil et al. [21], the urethral orifice was separate from the glans. Our technique was specifically adapted to accommodate the urethral anomaly encountered in this case. Although concurrent vaginoplasty and clitoroplasty could have been performed in a single operative session, the family elected to proceed with clitoroplasty alone at this stage.

Recognised complications of clitoroplasty in CAH include recurrent clitoral enlargement or pain arising from incomplete reduction or post-pubertal androgenic stimulation of residual erectile tissue [8]. Proximal corporeal transection mitigates this risk. Clitoral atrophy, secondary to compromised glans vascularity, may be prevented by gentle tissue handling, use of magnification, and topical papaverine application, all of which were employed in this case.

Limitations: Long-term functional follow-up extending into sexual maturity was not available for this report, and adult outcomes therefore remain unknown.

## Conclusions

Clitoromegaly secondary to CAH is encountered with relative frequency in the Indian subcontinent; however, reluctance to pursue early surgical intervention persists in many communities, often resulting in delayed presentation with advanced virilization. Ventral nerve-sparing clitoroplasty, performed under magnification with intraoperative papaverine, is a safe and effective approach that reduces clitoral size while preserving neurovascular integrity and long-term sensory function. Enhanced public and community-level awareness of the safety and availability of this procedure is essential to promote timely referral and early definitive management.
